# Intervention development of a brief messaging intervention for a randomised controlled trial to improve diabetes treatment adherence in sub-Saharan Africa

**DOI:** 10.1186/s12889-020-10089-6

**Published:** 2021-01-15

**Authors:** Natalie Leon, Hazel Namadingo, Kirsty Bobrow, Sara Cooper, Amelia Crampin, Bruno Pauly, Naomi Levitt, Andrew Farmer

**Affiliations:** 1 South African Medical Research Council, Cape Town, South Africa; 2 Malawi Epidemiology and Intervention Research Unit, Lilongwe, Malawi; 3 Chronic Disease Initiative for Africa, University of Cape Town, Cape Town, South Africa; 4 London School of Hygiene and Tropical Medicine, London, United Kingdom; 5 Department of Diabetes and Endocrinology, Chris Hani Baragwanath Academic Hospital, Johannesburg, South Africa; 6 Nuffield Department of Primary Care Health Sciences, University of Oxford, Oxford, United Kingdom

**Keywords:** Mobile health (mHealth), Formative intervention development, Brief SMS text-messaging, Behaviour change theory, Treatment adherence, Type 2 diabetes, Qualitative research, Sub-Saharan Africa

## Abstract

**Background:**

Brief messaging interventions, including Short Message Service (SMS) text-messages, delivered via mobile device platforms, show promise to support and improve treatment adherence. To understand how these interventions work, and to facilitate transparency, we need clear descriptions of the intervention development process.

**Method:**

We describe and reflect on the process of designing and pretesting an evidence- and theory-informed brief messaging intervention, to improve diabetes treatment adherence in sub-Saharan Africa. We followed the stepwise approach recommended by the Medical Research Council, United Kingdom (MRC UK) Framework for Development and Evaluation of Complex Health Interventions and guidance for mobile health intervention development.

**Results:**

We used a four-phase, iterative approach that first generated primary and secondary evidence on the lived experience of diabetes, diabetes treatment services and mobile-phone use. Second, we designed a type 2 diabetes-specific, brief text-message library, building on our previous hypertension text-message library, as well as drawing on the primary and secondary data from phase one, and on expert opinion. We then mapped the brief text-messages onto behaviour change (COM-B) theoretical constructs. Third, we refined and finalised the newly developed brief text-message library through stakeholder consultation and translated it into three local languages. Finally, we piloted the intervention by pre-testing the automated delivery of the brief text-messages in the trial sites in Malawi and South Africa. The final SMS text Adherence suppoRt for people with type 2 diabetes (StAR2D) intervention was tested in a randomised controlled trial in Malawi and South Africa (trial registration: ISRCTN70768808).

**Conclusion:**

The complexity of public health interventions requires that we give more attention to intervention development work. Our documentation and reflection on the StAR2D intervention development process promotes transparency, replicability, assessment of intervention quality, and comparison with other studies.

**Supplementary Information:**

The online version contains supplementary material available at 10.1186/s12889-020-10089-6.

## Contribution to the literature

- To optimise the success and uptake of mobile health (mHealth) interventions, researchers are starting to identify the evidence and theoretical basis for developing mHealth interventions.
- We build on this work by describing and reflecting on the process of designing and pretesting an evidence- and theory-informed brief messaging intervention to improve diabetes treatment adherence in sub-Saharan Africa. This promotes transparency, replicability, assessment of intervention quality, and comparison with other studies.
- We showed that despite differences in the two sub-Saharan African settings, patients’ experiences and health care journeys were similar, which bodes well for future development of standard diabetes messaging across different contexts.

## Background

Type 2 diabetes is major global public health concern [1]. Low- and middle-income countries (LMICs) are disproportionally affected by the substantial and growing burden of premature morbidity and mortality associated with chronically elevated blood glucose levels [2, 3]. Health outcomes for people treated for type 2 diabetes could be substantially improved in sub-Saharan Africa, but failure to take medicine regularly to treat diabetes (described as non-adherence) has been identified as a major problem [1]. Reasons for not collecting or taking medications as intended are well documented and include psychological factors, lack of social support, low levels of health literacy, and interactions with the health care system that do not support self-management [1, 4–7].

Mobile health (mHealth) interventions delivered by brief, Short Message Service (SMS) text-message (referred to here as brief messaging) for targeted client communication has been recommended for health issues regarding sexual, reproductive, maternal, and newborn health [8] and for adherence to anti-retroviral treatment and smoking cessation initiatives [9]. Brief text messaging is showing promise for supporting adherence and health behaviour in a range of conditions [9, 10], including for diabetes [11–14]. However, impact is inconsistent across studies [9, 11, 12, 14, 15], prompting researchers to call for more robust, large-scale research on better ways to develop and test digital communication technology for treatment adherence for diabetes and other chronic disease [5, 9, 11, 12, 14, 16, 17]. This should include evidence- and theory-informed formative work on mHealth intervention design and development to enhance the credibility, quality, and relevance of mobile health interventions for diabetes support [5, 11, 12, 18].

An investigation to understand the drivers of intervention development processes concluded that intervention designers often find it challenging that intervention design does not necessarily proceed in a predictable and controlled manner from acquired knowledge, and that there is a need for more guidance [19, 20]. Many mHealth interventions aimed at promoting healthy behaviour do not show what evidence or theory underpinned the message content or timing [11, 12, 21], whether and how a user-centred design approach was used [22], or whether there was any pretesting of the intervention [23]; though this is changing [24]. Accessible and transparent documentation of brief message interventions can help unpack the ‘black-box’ of intervention development to allow for appraisal of the quality and credibility of the intervention, show how it is adapted for local context, allow replicability and comparability with other studies, and improve our understanding of how and why the intervention might work [25]. There is a growing body of literature showing how researchers are identifying the evidence and theoretical basis for developing interventions [16, 24–36], including for mHealth interventions for diabetes [37, 38]. We are building on this work by describing and reflecting on the process of designing and pretesting an evidence- and theory-informed brief messaging intervention to improve diabetes treatment adherence in sub-Saharan Africa.

## Methods

### Study approach

We report here on the year-long formative work to develop and refine the StAR2D intervention prior to experimental testing. Following the period of formative work, the SMS text Adherence suppoRt for people with type 2 diabetes (StAR2D) intervention was tested in a pragmatic randomised controlled trial (RCT) in Malawi and South Africa in 2018 (trial registration: ISRCTN70768808). The aim of the RCT was to test the effectiveness of sending brief, automated SMS text-messages for improving health outcomes and medication adherence in patients with type 2 diabetes, compared to an active control, as detailed in the published protocol [39]. The primary focus is on adherence to medical treatment (attending appointments, collecting medicine, taking medicine) with secondary focus on adjunct general health and wellbeing strategies (i.e., food and eating, physical activity, tobacco and alcohol, stress management).

Intervention design necessitates a coherent idea of the evidence and theory behind the proposed intervention, adaptation to local context, and pilot testing before proceeding to an experimental or quasi-experimental study [24, 25, 40, 41]. In this paper, we report only on the formative work to develop the STAR2D intervention. The aim of the StAR2D intervention development study was to ensure the final brief (SMS) text-message intervention was theory- and evidence-informed, relevant, and acceptable to the target audiences, and appropriately aligned with the organisation of clinic care at the trial sites. Intervention messages were meant to advise people about the benefits of their diabetes treatment and offer motivation and encouragement around lifestyle and use of medication. We followed a step-wise approach as recommended by the Medical Research Council, United Kingdom (MRC UK) Framework for Development and Evaluation of Complex Health Interventions [40, 41] and other studies that provided guidance on the steps for intervention development of evidence- and theory-informed mHealth interventions for behaviour change [21, 22, 25, 42]. The primary focus of this paper is to document a systematic, transparent approach to intervention development in the context of an intervention that was tested experimentally. A secondary, but important focus is on the evidence generated in each phase to illustrate its contribution to shaping the final intervention.

### Study setting

We identified two trial sites, one in Lilongwe, Malawi, and one in Cape Town, South Africa, both in urban/peri-urban sites with a high burden of type 2 diabetes, with free public sector health facilities, serving populations living in low socio-economic settlements.

### Data collection and analysis

For the intervention development study, data collection took place at these two trial sites, and in the initial stage, in a third, site in Johannesburg, South Africa. We conducted primary research to seek input from adult health care users with type 2 diabetes, as well as health care providers involved with diabetes health care (including primary care nurses, doctors, pharmacists, administrative support staff and health facility management). Secondary data sources were identified through literature searches and included document review of local diabetes policy and clinical guidelines. We did not conduct a full systematic literature review. Our pragmatic approach was a combination of identifying relevant systematic reviews to start with, hand-searching of relevant papers in systematic reviews, and a general targeted literature search for key articles.

**Fig. 1:**
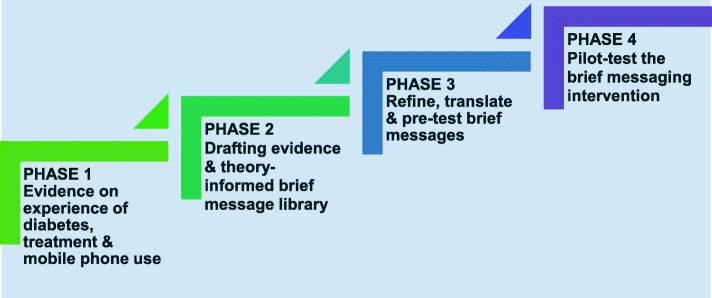
The four phases of the StAR2D intervention development process

We followed four phases of intervention development (as shown in Fig. 1). For each of the four phases, we describe the aim and methodological approach, provide a summary of the results that emerged from that phase, and reflect on how this contributed to shaping the content of the intervention. For this description and reflection, we draw on the primary and secondary data collected in the four phases, as well as on our operational research documentation (protocols, operational plans, project management meeting notes and discussions).

We used multiple, qualitative research strategies over a 12-month period of intervention development (2016–17). In the Results section, we provide an overview of the methods for each phase, as this is part of our intervention description. For ease of reading, we provide an overall summary of the data sources for the formative work.

**Phase 1:**

- We drew on evidence from the secondary data sources (literature and document reviews) and primary research.
- We conducted patient focus group discussions (FGDs) with adults with type 2 diabetes (12 FGDs, 89 participants in total, 58% female):
- Lilongwe, Malawi: three single gender FGDs, two with males (14 participants, age range 28–78 years) and one with females (7 participants, age range 19–63 years)
- Cape Town, South Africa: six mixed gendered and 1 one female FGDs (8 male and 32 female participants, age range 47–80 years) *
- Johannesburg, South Africa: one male FGD (15 participants, age 21–70 years) and one female FGD (13 participants, age 42–68 years)
- Field observation was done to map the organisation of health services and patient care pathways for diabetes treatment in both trial sites.

**Phase 2:**

Our data sources for this phase were:

- SMS library from the previous hypertension adherence study
- Data collected in Phase 1 (primary, secondary and mapping exercise)
- COM-B behaviour change taxonomy: We used theory-informed behaviour change constructs to guide the content of the messages as proposed by the Capability, Opportunity, Motivation-Behaviour (COM-B) theory [43–45]. The COM-B framework is widely used for studying behaviour change in adherence, has the benefit of consolidating evidence from a range of other behaviour theories, and can account for contextual factors (like socio-economic conditions and health service factors) [43, 45–47].

**Phase 3:**

We conducted FGDs with adults with type 2 diabetes (6 FGDs, 56 participants in total, 59% female):

- Lilongwe, Malawi: two male FGDs (16 participants, age range 29–68 years), and two female FGDs (16 participants, age range 38–72 years). Time since their diabetes diagnosis ranged from 1 month to 16 years.
- Cape Town, South Africa: two mixed gender FGDs (7 male and 17 female participants, age range 38–71 years). We ensured a mix of people from the two main language groups in the catchment area (isiXhosa and Afrikaans). *
- We conducted in-depth qualitative interviews (IDIs) with 11 patients and 18 health care facility staff in the trial sites.
- We conducted five consultations with experts external to the trial team, including in diabetes health promotion (3), nutrition (1), and exercise (1), and we asked the clinical experts in trial team to review the message content, language, and tone.

**Phase 4:**

- For the pilot testing, we conducted brief (less than 5 min) weekly telephone interviews with 10 patients in each site over a 3-week period to check the technical delivery of the intervention and how messages were understood.

* (There are gaps in data on age and time since diagnosis as this information was not consistently collected).

We chose to use both in-depth interviews and focus group discussions as combining these methods can facilitate a deeper comparison of perspectives, improve data completeness, and enhance the trustworthiness of findings [48]. While interviews enable one to explore individual views and experiences in detail, the conversational nature and interaction in a focus group can help generate additional insights, test and refine understandings gained in individual interviews, and stimulate commentary that may not have been elicited from individual interviews [49]. See Additional file 1 for the interview guide used for individual interviews and focus groups conducted in phase 1.

In Phase 1 and 3, patient participants were selected through a combination of purposive and convenience sampling. In purposive sampling, we aimed to recruit a diverse group of participants that resemble the diabetes patient population in terms of gender, age, and duration of diabetes. Convenience sampling was done with the help of nurses alerting patients to the study and recruitment of participants in waiting rooms. For the pilot testing in phase 4, we recruited from participants from phase 3. Staff participants were purposively selected and included staff and management associated with delivery of the diabetes service, including facility management, clinical staff, and health promoters. Two health experts (nutrition and exercise) and two health promotion experts (one in Malawi and one in Cape Town) participated. They were recruited based on recommendations from other health providers and people in the trial research team.

Data analysis for the primary data collected in phase 1 was analysed using a thematic analysis approach. Focus group and individual interviews were audio recorded, transcribed, and coded by one researcher (NLn) using a set of codes we developed based on the research question, interview schedule, and initial themes emerging from the early interviews. As a first step in the analysis, the researchers compiled an interview summary within 36 h of conducting the interviews and focus groups to summarise the main content and provide researcher reflections. These were used for initial analysis in the iterative data analysis process. Analysis of data collected in phase 3 and 4 focussed on identifying key issues and recommendations to consider in refining the draft diabetes text message library. Standard approaches to ensuring the quality of the methodology were used, including the use of a coding framework and review of the final themes by researchers who conducted the data collection.

**Fig. 2:**
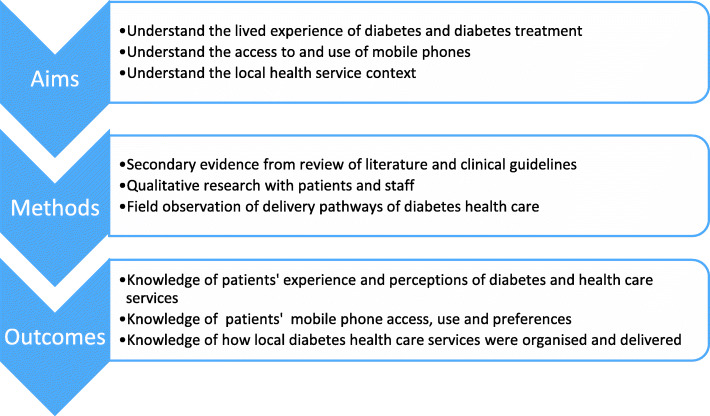
Phase 1: aims, methods and outcomes

**Fig. 3:**
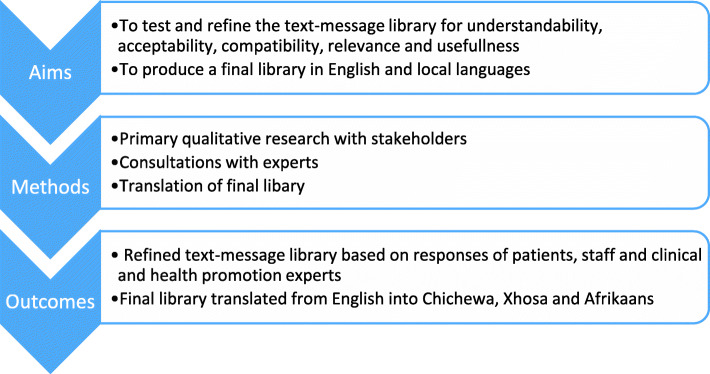
Phase 3: aims, methods and outcomes

**Fig. 4:**
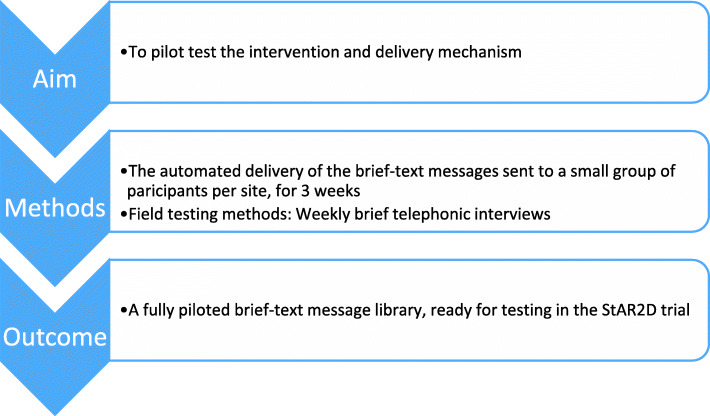
Phase 4: aim, methods and outcomes

For this description and reflective analysis, we draw on the data described above. We also draw on our project documentation (operational plans, meeting minutes, interim reports), and reflect on our formative work, almost as if we are participant observers of our own processes. In the Results section, we provide an overview of the methods per phase (in Figs. 2, 3, and 4), and in the narrative, we highlight key findings and reflect on issues that shaped the intervention development. Findings from elements of the primary qualitative data have been published elsewhere [50].

### Ethical approval

The protocol was approved by the University of Oxford Tropical Research Ethics Committee (OXTREC, ref.: 22-15), the University of Cape Town Research Ethics Committee (UCT HREC, REF: 126/2015), the University of Witwatersrand Research Ethics Committee (R14/49) and the Malawi National Health Services Research Committee (NHSRC #15/7/1425). In addition, the relevant health authorities granted permission for the study, including the intervention development work. All participants who were included in the formative study provided written, informed consent to participate.

## Results

### The intervention development process: designing, refining, pre-testing and piloting of the StAR2D intervention

We employed a stepwise and iterative process for the intervention development (or formative) study, as illustrated in Fig. 1. To summarise: first, we identified diabetes patients’ experiences and perceptions of diabetes and treatment, and then we used this evidence to build on a previous set of text-messages we had designed for a trial on blood pressure control [26, 51]. Through a process of consultation with stakeholders (patients, staff, StAR2D trial research team members, health promotion experts), and using the primary and secondary evidence we collected, we designed a draft brief text-message library with 222 content messages. Through further consultation with stakeholders, we finalised a library of 156 brief text-messages (and 16 trial-related messages) and translated it into three local languages.

We used theory-informed behaviour change constructs to guide the content of the messages, as proposed by the Capability, Opportunity, Motivation-Behaviour (COM-B) theory [43–46]. The COM-B theory states that an individual needs adequate capability, opportunity, and motivation for a behaviour to take place (such as taking medicine), and that a deficit in any of these three areas means the behaviour is unlikely to occur. An individual’s capability to adhere may be affected by psychological factors of knowledge and memory, opportunity may be affected by physical and social barriers to access medicine and support, and motivation may be affected by psychological factors such a self-confidence, values and beliefs [52].

Finally, we pilot-tested the automated, brief (SMS) text delivery system with the intended target audience before handing over the final StAR2D brief text-message library to be tested in the StAR2D RCT study. Below, we provide a detailed description of, and reflection on, each of the four phases shown in Fig. 1. In Additional File 2, we provide a TIDier checklist with a summary description of the final StAR2D intervention that was experimentally tested [39]

.

#### Phase 1: evidence on experiences and perceptions of diabetes, diabetes treatment and mobile phone use

To inform the content of the SMS brief text-messages and other delivery components (timing, frequency, language preferences), we gathered information from secondary sources (systematic and literature reviews and clinical guidelines) for pertinent information on patient’s experience of diabetes and adherence. We conducted primary research on experiences and perceptions of diabetes, diabetes care, and treatment services with patients and staff. We explored patient access, use of mobile phones, and attitudes to mobile phone messaging. Through field observation and staff interviews in local clinics, we mapped the provision of diabetes care services at the two trial sites to allow the tailoring of messages to the local service delivery context. As shown in Fig. 2, we summarise the aims, methods, and outcomes of the first phase of the intervention development study.

##### Generating evidence on experiences of diabetes, treatment and mobile phone use

In Malawi, patient participants were a mix of urban and rural, which contrasted with predominantly urban participants in South Africa. Nevertheless, patient participants across the country settings identified similar experiences, struggles, and needs. Many experienced their diabetes diagnosis with shock and disbelief at first. Participants felt supported by family and their faith, which helped them come to terms with and cope better with the illness. Generally, participants understood and accepted the need for long-term medical treatment, but they had lingering questions about the underlying reasons for their disease, and uncertainties about how best to manage their illness.

For the most part, participants described themselves as adherent in terms of picking up their medicine as required and taking the medicine as prescribed. Some acknowledged that adherence was an ongoing struggle for them. Several patients took pride in themselves for being ‘good’ patients when it came to taking their medicine as prescribed. However, when probing, there was a wide spectrum of understanding and practice of what constituted “good” adherence. Both sets of participants (those who reported being adherent and less than adherent) identified a range of personal, social, economic, health system struggles, including family and socio-economic stressors. Situations that posed a risk (for disrupting normal patterns of eating and taking of medication) were substance misuse, social engagements (family or other social gatherings and festivities) and travelling. Experiences with the health services also posed a risk to adherence. Issues included medicine stock-outs, long waiting times and difficult patient-staff interactions.

For the most part, men and women had similar experiences of living with diabetes. One gendered difference we observed was the dependence of some men on their female partners. These men noted that their female partners were their main source of social and emotional support in coping with diabetes (including helping them to eat more healthily). Another related, but more stark gendered difference was that several men (women also, but more so in men) expressed concerns about the effect of diabetes on their sexual health and well-being. The men feared that sexual dysfunction could jeopardise their intimate partnerships and also threaten their main source of social support. Details of this gendered concern about sexual well-being are described elsewhere [50]. We refrained from including messaging on sexual function and diabetes as we thought the topic was too complex to address through the medium of a text message.

Participants reported ongoing struggles with accessing appropriate health information. It was especially hard for them to find practical information about healthy living that fit with their lives and poor socio-economic conditions. They asked for text-messages to include practical, locally relevant advice on healthy eating and keeping active. Where feasible, these issues were addressed in the text messaging in various ways, as discussed in Phase 2 below.

We examined mobile phone access and use to explore the feasibility and acceptability of sending patients brief text messaging in support of medication use for diabetes. Participants had access to either their own or a shared phone (a mixture of basic and smart phones) and could operate these devices by themselves (or with the help of family) to receive and send messages, and everyone used some form of SMS text messaging. For those with smart phones, the cost of internet access was often prohibitive. They were comfortable with the idea of receiving health promotion-related messaging on their phone but wanted a way to easily distinguish health messaging from spam messages. In particular, they asked that the message sender be identified as coming from the health services to avoid it being deleted as spam. They wanted the option of receiving messages in their first language.

The patient flow and treatment pathways were studied by observing how the diabetes services were delivered at each trial site. This provided us with locally relevant data that could be used to tailor the messages for each site regarding frequency of medication pickup and medical appointment, as well as the expected patient flow and treatment pathways from entering to exiting the facility.

#### Phase 2: drafting the evidence and theory-informed brief text-message library

In Phase 2, we designed a draft set of brief text-messages suitable for supporting adherence to diabetes care. This involved three steps. The first was adapting a previous set of text-messages on hypertension adherence. This set was also developed with patient and provider input [26]. The second step was to craft new diabetes-specific messages drawing on the primary and secondary evidence on diabetes and mobile phone experience that we generated in Phase 1. Thirdly, we mapped the messages onto known behaviour change strategies. Table 1 shows the structure, topics, and examples of the final brief text-message library. It consists of 156 diabetes content-related messages and 16 trial-related message. The main components are enhancing primary adherence (medical treatment) and enhancing secondary adherence (general health and well-being). We decided a-priori on the ratio of messages, with most of the messages focussing on supporting medication use, in line with the primary focus of the trial.

##### Message content development

Many health risk behaviours for high blood pressure (HBP) and type 2 diabetes are shared, so messages in the blood pressure study that were broadly applicable to both were reviewed and retained. Many people with type 2 diabetes also have comorbid HPB, so messages that would be helpful for this, and for general adherence, were also retained.

**Table 1:** StAR2 brief text-message library: structure and examples

**A: ENHANCE MEDICAL ADHERENCE**	Number of SMSs
**1. Medication collection** Example: *“We think it might be nearly time for your next medicine date. Please check your clinic card and come on your date. Thnx [DiabetesTeam@Van Clinic]”*	22
**2. Medical appointment** Example: *“Attending clinic appointments will help you stay healthy. Pls plan ahead for your arrangements (travel, work, childcare). Thnx [DiabetesTeam@Van Clinic]”*	19
**3. Taking medication regularly** Example: *“Taking your diabetes and other meds as prescribed means that you are in control of your health. Keep it up! Thnx [DiabetesTeam@Van Clinic]”*	48
**4. Medical adherence support: general** Example: *“It’s easy to forget to take your meds at social occasions. Keeping some meds in your wallet or pocket could help. Thnx [DiabetesTeam@Van Clinic]”*	18
Subtotal	107
**B: ENHANCE GENERAL HEALTH AND WELL-BEING**	
**1. General health and well-being** Example: *“Taking your meds, exercising and eating healthy can improve your blood sugar levels and prevent or reduce complications. Thnx [DiabetesTeam@VanCHC]”*	27
**2. Exercise, nutrition, and health** Example: “*Being more active can help you feel more energetic. Walking, standing, jumping are healthier than sitting. Thnx [DiabetesTeam@Van Clinic]”*; *“Healthier eating includes eating a variety of protein (beans, meat, fish and eggs), more vegetables and less starchy foods. Thnx [DiabetesTeam@VanCHC]”*	9
**3. Smoking and drinking** Example: *“Smoking increases your risk of hypertension, heart disease & stroke, so it’s better to avoid smoking or cut down. Thnx [DiabetesTeam@Van Clinic]”*	6
**4. Stress management** Example: *“Coping with diabetes and a busy life can be stressful. Learning to manage your stress better can help improve your health. Thnx [DiabetesTeam@Van Clinic]”*	7
Subtotal	49
**TOTAL**	**156**
C: TRIAL-RELATED AND GENERAL	16

Drawing on the literature, clinical guidelines, and input from participants (patients, staff and health promotion experts), we designed additional brief messages. In phase 1, participants identified a range of challenges to being adherent and raised the need for information, encouragement, and practical advice. Where feasible, the message content we developed addressed these informational and support needs, including acknowledging stressors and providing encouragement, affirming patient efforts, and encouraging self-efficacy. Besides the need for reminders to pick up and take medicine, messages aimed to increase awareness of the long-term nature of the disease, the need to prevent complications, and the importance of healthy living.

Participants specifically asked for information and advice to help them have a healthier lifestyle. They wanted to know what ingredients they could substitute for the regular food items they consumed (those food items they considered unhealthy) and how to measure the right portions/quantities for meals. With the help of a nutritionist with expertise in behaviour change, and drawing on local health promotion materials, we designed messages about healthy nutritional options, considering the local context. Messages gave advice, amongst others, on how to cut down on potentially unhealthy snacks (such as sweets and sugary drinks), substituting healthier alternatives for flavourings like salt, finding healthier local alternative foods, healthier food preparation, eating a balanced diet, and limiting portion sizes. We included messages on ways to limit the high-risk situations participants had identified (such as social events and travelling). Message content aimed not only to provide active reminders and practical information, but also to instil a sense of hope and self-responsibility. Several messages focussed on encouragement, motivation, and providing opportunity for initiating positive behaviour change. These included messages that acknowledged their life stressors as well as their frustrations with the health services, and messages encouraged them to view the health service as a partner in managing their disease.

We categorised the final set of 156 messages into 90 ‘core’ messages. These were messages that had unique content and expressed a key idea or behaviour change mechanism (e.g. shaping knowledge, cue to social support, practical reminders or lifestyle advice). The rest were variations on those core messages. We had a sufficient quantity and variation of messages to send regular appointment and prescription pick-up reminders (these were standard and repeated), as well as additional messages using a frequency not more than 4 times per week, sent on random days. Participants had the option of not receiving a message on at least one day of the week to suit their preference. For instance, some participants did not want to receive messages on days that they practiced religious observances, and messages could be tailored to such individual patient preferences.

##### Mapping messages to represent behaviour change techniques

To increase the chance of the messages having the desired positive effect on adherence behaviour, we wanted the messages to reflect directly, or indirectly, a known behaviour change strategy [29, 30, 45–47]. This would also later assist our process evaluation after the trial, where we intended to examine the potential causal pathways that may explain patient behaviour change (or lack thereof). Table 2 shows examples of how each message was mapped onto a behaviour change technique.

We examined each message for its underlying intention (by asking what kind of change the message intended to produce, and through what mechanism), and then categorised messages under the three key components of the COM-B behaviour change theory constructs which focusses on capability, opportunity, and motivation factors for changing behaviour [43, 44]. We refined this by allocating messages to one of the 16 most common clusters of behaviour change techniques identified by Michie et al. [43]. The mapping was done in stages, first by one researcher (KB) who mapped the whole library. This was reviewed by the lead researcher (NLn) and another (SC), sitting together. We balanced the number of messages in each of the key behaviour change domains (Capability, Opportunity, Motivation) and resolved disagreements by consensus among the 3 researchers.

**Table 2:** Examples of text-messages mapped onto COM-B behaviour change techniques

Brief text message	COM-B behaviour change domain and techniques
*Struggling to remember to pick up your meds? A trusted friend or family member could help remind you. Thnx [DiabetesTeam@VanClinic]*	CAPABILITY Social Support – cue to action
*Your good health is important. Pls take your meds as prescribed for all your health problems, even if you feel fine. Thnx [DiabetesTeam@VanClinic]*	MOTIVATION Addressing known triggers for sub-optimal adherence
*Attending clinic appointments will help you stay healthy. Pls try to plan ahead for your arrangements (*e.g. *travel, work, child care). Thnx [DiabetesTeam@VanClinic]*	OPPORTUNITY Enhancing physical opportunity (mobilise time and resources)
*It’s easy to forget your meds at home when travelling. Pls remember to take your meds along on your trip. Thnx [DiabetesTeam@VanClinic]*	CAPABILITY Building flexible knowledge of strategies to have medicines available to take
*Well-controlled blood sugar (taking your meds & a healthy lifestyle) helps decrease the risk of a stroke. Thnx [DiabetesTeam@VanClinic]*	CAPABILITY Psychological capability - knowledge of natural disease progression
*Caring for your feet is really important. Ask us for information on how to look after your feet. Thnx [DiabetesTeam@VanClinic]*	CAPABILITY Enhancing psychological capability for self-management
*Sugar diabetes can affect anyone. Men, women & people of all ages, in all countries are living with sugar diabetes. Thnx [DiabetesTeam@VanClinic]*	OPPORTUNITY Social opportunity - encouraging social norms that disease is not due to personal failing
*It can be difficult to change what you eat. Try to make a few small changes to your diet that you can stick to. Thnx [DiabetesTeam@VanClinic]*	OPPORTUNITY Social opportunity -cue to action
*Healthy food is not always expensive. Beans and eggs provide good protein and cost less than meat. Thnx []DiabetesTeam@VanClinic]*	MOTIVATION Reflection, evaluation and challenging automatic processes
*Losing weight can be hard. Start with small steps, like eating a fruit for a snack instead of biscuits or sweets. Thnx [Dr@VanClinic]*	OPPORTUNITY Social opportunity - cue to action

#### Phase 3: refine, pre-test and translate brief text-messages

##### Message pre-testing

As outlined in Fig. 3, we tested and further refined the brief text-messages through primary research with stakeholders and expert consultation. We purposively selected a sample of 15 messages for in-depth testing in patient focus groups and staff interviews. The selection criteria were informed by our need to investigate issues of language and tone, understandability, compatibility, acceptability, relevance, and usefulness. We asked participants to review the appropriateness of the translated versions of the 15 messages. We explored and refined our assumptions about the underlying behaviour change mechanisms of these messages, using cognitive interviewing techniques (often used in market research) [53] by asking participants what they understood by each message, and what reactions the messages elicited (in terms thoughts, feelings, and potential behaviour and action). Below, we highlight a few key issues we addressed in refining the message and delivery mechanisms.

##### Message tone

In both country settings, patient participants shared similar sentiments about the importance of striking the correct message tone. They wanted encouragement, hope and practical advice, provided in a tone that is supportive, kind, polite, and respectful. There was a need for more emotionally supportive messaging that recognised their frustrations and stressors. They explained that they would feel discouraged by messages with an authoritarian or too directive a tone (though some felt a directive tone was acceptable for messages about medication adherence and conveying warnings about diabetes complications). This contrasted with staff preferences for more directive messages to emphasise the importance following professional advice. For example, staff preferred the use of a directive tone, such as “*You must*” as in, “*You must remember to pick up your medication*”. We took care to balance this patient-staff tension and opted for a more motivational tone that patients approved of, and that was in line with the motivational element of the COM-B behaviour changes strategies. For example, we often posed a question or explanation before offering advice, as in, “*Do you have a clinic appointment this month? Then please do not miss it. Thnx (DiabetesTeam@Van Clinic)*” and, “*To keep your blood sugar levels under control, please keep taking your meds as prescribed. Thnx (DiabetesTeam@Van Clinic)*”.

##### Message credibility

We ensured each message had a sign-off that clearly identified the sender as part of the health team at the local clinic, for example *“[DiabetesTeam@Van Clinic]”*. We did not use the names of providers. While some thought using the name of a real doctor is a sign of personalised care, others worried that staff may take offence and regard patients as being too demanding if they asked for the named doctor. Participants noted that if messages were to be believable, they should reflect the reality of their (the patients’) real experience and perceptions of the health service, and not an idealised view. For example, they did not want messages to convey an idealised, exaggerated view of the responsiveness of health staff, but rather and acknowledgement of issues that frustrated them about the health service.

##### Local adaptation and translation of messages

The similarities in the patient and staff responses across the sites meant we could standardise the core content and number of messages across sites. This still required adjustment of core messages to fit better with local context, for example, reflecting local food options, preferences, and lifestyle practices. Once the content of the 156 brief text-messages in the StAR2D main library was finalised, we used professional translators for the isiXhosa and Afrikaans translations in South Africa and local research staff for the Chichewa translation in Malawi. Further adaptations were required during the translation to consider cultural nuances in local languages (and given the 160-character limit for SMS messages). User feedback was critical in guiding this. A health promotion expert in both sites checked the quality and appropriateness of the final messages.

##### Delivery mechanisms and tailoring

Participants in the intervention group received brief trial-related messaging and three to four automated health text-messages per week on their mobile phone for 12 months, free of charge. Participants in the active control group would receive brief trial-related and infrequent (one every 6 weeks) non-health related messages. The 12-month delivery period is based on evidence recommending intervention duration longer than 6 months [12]. The research team used their experience with a prior study [5, 26, 51], and recommendations from the literature, to set the frequency and timing of messages to achieve a good dose response and avoid message fatigue [28, 30]. We tailored the reminder messages to the service organisation in each site, such as the frequency of medication pick-ups.

Message delivery was one-directional (did not allow for a response from participants), a design feature that was considered important to enhance the feasibility of the intervention within a resource-constrained setting. Participants could preselect and change the language of preference and select preferred times for the delivery of the messages via the automated digital platform.

#### Phase 4: pilot testing the text-message intervention and delivery mechanisms

We piloted-tested the automated delivery of the brief text messaging before finalising the StAR2D intervention, as shown in Fig. 4. (For details of the final StAR2D intervention, see the TIDier checklist in Additional File 2). The StAR2D main library of messages was loaded onto an automated text delivery platform using open-source software (Open Medical Records System (OpenMRS.org)), with secure information exchange protocols. We used a third-party bulk SMS-delivery provider. Data was transmitted using a low-cost mobile phone linked to Sana Mobile (MIT)), for the data collection system.

The pilot testing allowed for final checks and fine-tuning of the technical delivery of the automated messaging system before the intervention was ready to be tested experimentally in the StAR2D trial. The automated digital delivery mechanism for this intervention was not integrated to local electronic information systems, as this was not feasible. In both sites, electronic information systems were not fully operational.

To pilot test the delivery mechanisms, we signed up 10 participants in each site, and sent them an automated sample of SMS brief text-messages in their preferred language on their own mobile phone for a period of 3 weeks. We did weekly brief telephone interviews (less than 5 min) with pilot-test participants to check if they received the messages as intended (the days, time, number of messages per week, in their chosen language) and could open it in readable format. We also checked their reaction to the message - how they understood and interpreted it. We did not get any feedback on message content that required us to make further changes. Technical problems with the selection of preferred language and message delivery time were picked up and corrected. The end of the piloting phase concluded the intervention development research phase, and the intervention was now ready to be tested experimentally in the StAR2D trial. The TiDIER checklist in the Additional file 2 provides an overview of the intervention components.

## Discussion

This paper describes and reflects on a systematic and iterative approach to the development of a brief text-message intervention that was experimentally tested in a randomised controlled trial to improve diabetes treatment adherence in sub-Saharan Africa.

We followed the steps recommended by MRC UK framework for developing and evaluating complex health interventions [40, 41], and followed guidance for mobile phone-based brief text-message development elsewhere [16, 21, 22, 24, 34] aimed at promoting good intervention design prior to experimental testing. This was done by gathering evidence to inform the design, applying theory to strengthen the design, getting user-feedback on acceptability and relevance, and testing feasibility through piloting, as shown in other study interventions [24–29, 32, 33, 35, 37, 38, 54].

The step-wise methodology contributes to a growing body of literature illustrating the value of following a detailed and systematic approach to intervention development [12, 24]. The study builds on our previous research of developing and testing (through an RCT) a brief-messaging intervention for adherence to hypertension treatment in South Africa [26, 51]. By documenting our formative work, we were able to illustrate the step-wise approach we took to shaping the intervention and bring transparency to intervention development process, thus unpacking the ‘black box’ [25] of our intervention development.

Our study is the first to systematically document the development of a brief diabetes-related text-message library for supporting adherence across two sub-Saharan sites, using multiple data sources, and a large data set that is representative of both genders. Similar studies have been conducted for diabetes in the US [37] and New Zealand [55], but with more focus on tailoring to patient preferences and clinical characteristics. The New Zealand Self-Management Support for Blood Glucose (SMS4BG) intervention included similar prompts around diabetes education, management, and lifestyle factors (healthy eating, exercise, and stress management). It was also unidirectional (send-only), except for the blood glucose monitoring reminders, which allowed for interaction around blood glucose test results [55]. The New Zealand study showed a modest improvement in glycaemic levels in adults with poorly controlled diabetes [13] that was sustained two years after randomisation [56].

The work of intervention development does not occur in a vacuum though. There are tensions between the goals of the developers to design an intervention that can improve health care within a real world setting, while considering the limited resources of the health care context and the research funding context [19, 20]. In this study, we balanced several tensions in crafting our message content and tone. For example, we prioritised medical adherence (taking of medicines as prescribed and medical follow-up), while acknowledging the importance of healthy lifestyle (secondary) adherence. We balanced patient and staff perspectives, recognising the interconnected nature of patient-staff interaction. We grappled with how best to convey (sometimes complex) health messaging, within a SMS 160-character limit, and how to do appropriate message translation. We also had to manage different perspectives among the research team on how best to strike the balance between these tensions. Finally, we needed to strike a balance between design features that could enhance the effectiveness, and features that could enhance the feasibility and potential upscale-ability in low-resource settings. For instance, in the StAR2D intervention design, we reduced the technical complexity by limiting the personalising of patient, clinical, and medication characteristics, to make the intervention more feasible for implementing in a low-resource setting.

We showed that despite differences in country and health system contexts, patients in two sub-Saharan settings had similar experiences of diabetes disease and of their health care journey. Patient participants identified a range of personal, social, and health care factors that pose a risk to their ability to effectively manage their disease. They also identified the support they got from family and friends, and from their faith. Similar factors have been documented elsewhere [1, 4–7, 37, 57, 58]. In both settings, participants wanted messages to be congruent with their real world experience of health care and acknowledge the social and economic challenges they faced.

As in other studies, participants identified factors that may influence patient engagement with a mHealth brief messaging program. These include receiving information from a credible source, consistent with medical advice, that provides encouragement and motivation, presented in a polite and respectful tone [5, 24, 29, 30, 32, 51, 59]. The similarities across settings in sub-Saharan Africa bodes well for developing standard diabetes messaging across different contexts [5], but messages still needed to be adapted for local language and local food and cultural practices.

In our reporting, we address some, but not all of the WHO mHealth evidence reporting and assessment (mERA) guidelines aimed at improving completeness of mHealth intervention reporting [60]. Chiefly, we described the development of the content, delivery and technical platform, user feedback, content testing, adaptations to local context. We noted the challenge of interoperability with routine information systems, a common barrier for scalability of mHealth interventions [61]. This detailed description of the development of our intervention in a low resource setting can go some way to support replicability of the intervention development processes elsewhere, including in other low resource settings.

### Limitations

Using theory to design effective digital communication interventions is an ongoing challenge and superficial use of behaviour change theory is an obstacle to effective intervention design [21, 62]. Our challenge was how to develop depth in theoretical underpinning of the messages, and how to measure the ‘active ingredients’ of text-messages aimed at supporting adherence behaviour [21, 62]. The extent to which the messages will elicit the intended cognitive, emotional, and behavioural reactions remains uncertain [12, 18, 63, 64].. Future research may usefully explore effective ways of infusing behaviour change theory into targeted patient digital communication, including how to effectively evaluate the underlying causal mechanisms and identify the ‘active’ ingredients of change associated with digital targeted communication using text-messaging [12, 18, 21, 24, 62–64]. This could include work to validate a set of brief diabetes-related health messages, for its applicability across LMIC settings (and for conditions other than diabetes), as was done in the Latin-American context [27]. Further, studies should explore the relationship between well-designed, evidence- and theory-informed interventions, and intervention successes, where success includes the extent to which the intervention can be scaled up to a health system-wide level [8, 11, 12, 24]. Finally, sampling approaches might have introduced bias, and the findings might not be representative of all people with diabetes living in the study areas in Malawi and South Africa.

## Conclusion

The complexity of public health interventions requires that we pay more attention to intervention development work. Our documentation and reflection on the StAR2D intervention development process promotes transparency, replicability, assessment of intervention quality, and comparison with other studies.

## Supplementary Information

**Additional file 1: Interview guide.** Interview guide used for the in-depth individual interviews and focus groups with patient participants in Phase 1.

**Additional file 2: TIDIER Checklist.** A summary description of the final StAR2D intervention that was experimentally tested.

## Data Availability

The datasets that support the findings of this study are available from Dr. Natalie Leon (author NLn), but restrictions apply to the availability of these data, which were used under license for the current study, and so are not publicly available. Data are however available from the authors upon reasonable request and with permission of Dr. Leon and Prof Andrew Farmer.

## Abbreviations

SMS: Short Message Service
MRC UK: Medical Research Council, United Kingdom
mHealth: Mobile health
StAR2D: SMS text Adherence suppoRt for people with type 2 diabetes
LMICs: Low and middle-income countries
COM-B: Capability, Opportunity, Motivation-Behaviour
HBP: High blood pressure
