# Supplementary material for: Intervention development of a brief messaging intervention for a randomised controlled trial to improve diabetes treatment adherence in sub-Saharan Africa
Source: BMC Public Health. 2021 Jan 15;21:147. doi: 10.1186/s12889-020-10089-6 (PMC7811237; doi:10.1186/s12889-020-10089-6)
Supplement: Supplementary file 2 — Additional file 2: TIDIER Checklist. A summary description of the final StAR2D intervention that was experimentally tested. [file 12889_2020_10089_MOESM2_ESM.docx]

| **TIDieR checklist for the SMS supporting treatment for people with type 2 diabetes (StAR2D) intervention** | | | | |
| --- | --- | --- | --- | --- |
| **1.** | **Brief name** | SMS text Adherence suppoRt for people with type 2 diabetes (StAR2D) a brief messaging intervention for treatment adherence support delivered by SMS text-message | | |
|  |  | Active Control Group | Intervention Group | |
| **2.** | **Rationale or theory** | Treatment for patients with type 2 diabetes could be substantially improved in sub-Saharan Africa. Amongst the problems identified, failure to take medicines to treat diabetes regularly is a major problem. Mobile phones are widely available in these settings including among people with diabetes and linked technologies such as SMS-text messaging, have shown promise in delivering low cost interventions efficiently.  Brief messages, contextualised to place, time and person are one medium through which interventions to support treatment adherence can be delivered. A process of developing such messages guided by a rigorous theory of behaviour change is likely to be helpful in understanding and evaluating implementation of content for digital health technology.  B-COM theory and associated behaviour change techniques used to guide brief message development with an explicit strategy of addressing aspects of treatment adherence known to be important in management of chronic diseases of lifestyle; primary focus on adherence to medical treatment (attending appointments, collecting medicine, taking medicine) with supplementary focus on adjunct general health and wellbeing strategies (i.e. food and eating, physical activity, tobacco and alcohol, stress management) based on findings from formative work on how to make intervention more credible and acceptable. The decision to have an active control group is based on published evidence in the field. The timing and number of messages based on literature and evidence from formative work aimed at establishing the minimum number of messages perceived by end-user as "regular". | | |
|  |  | Active control: Infrequent non-health related messages sent to all participants,   1. Maintain participant interest in the trial. 2. Make it less clear who was getting which intervention 3. Exclude receipt of “any SMS” as effecting health-related behaviour | Brief messaging intervention:   1. Timely, relevant, personalised information designed to address common challenges to treatment adherence (medicine and lifestyle) 2. Message content developed using local and international guidelines with input from diabetes, primary care and public health physicians, dieticians and sports medicine researchers 3. Content split 70% on adherence to medicine with an additional 30% of messages focussed on supporting healthy lifestyle choices 4. Core messages with variations to enable thematic repetition but limit message fatigue and boredom 5. Messages delivery frequency and timing based on review of literature and expert opinion 6. Most messages unidirectional, automated bi-directional options for language or time of message change 7. Messages designed to be polite, direct, signed off by the health team. | |
| **3.** | **Materials** | Available health materials on type 2 diabetes provided by the health care service also made available to study participants | | |
| **4.** | **Procedures** | 1. Language and timing of messages selected by participant 2. Types of brief messages 3. Welcome message (confirming sign-up) 4. Message with options for changing delivery time or language of messages 5. Non-health related messages sent every 6 weeks for 12-months 6. Congratulatory message on participant's birthday 7. Thank you message at end of study | | 1. Language and timing of messages selected by participant 2. Types of brief messages 3. Welcome message (confirming sign-up) 4. Message with options for changing delivery time or language of message 5. Non-health related sent at 6-weekly intervals 6. Congratulatory message on participant's birthday 7. Brief treatment and lifestyle adherence support messages randomly selected from library (with rules that ensured individual messages were not repeated) and sent several times per week, each week for 12-months 8. Appointment reminder and follow-up messages sent 48 hours before and after scheduled appointment (and strategies for rescheduling appointment ‘s no longer convenient or have been missed 9. Thank you message at end of study |
| **5.** | **Intervention provider** | Automated SMS text-message delivery platform using open-source software and third party bulk SMS-delivery provider | | |
| **6.** | **Modes of delivery** | Intervention delivered via 160 characters SMS-text sent to individual participant’s own handset. Initial message sent to all enrolled participants is Welcome message confirming sign-up, thereafter after messages sent in automated fashion to individual participants as per randomised allocation for 12-months. No messages sent on Sundays (results from formative research) | | |
| **7.** | **Location where intervention occurred** | Community | | |
| **8.** | **Number of times intervention was delivered over what time period** | Brief trial related and non-health messages sent once every 6 weeks for 12-months | | Brief messages sent three (min) to four (max) times per week for 12-months |
| **9.** | **What, why, when, how intervention was personalised or adapted** | 1. Language and timing of messages selected by participant 2. Date of birth recorded for birthday message (as self-reported at enrolment) | | 1. Language and timing of messages selected by participant 2. Date of birth recorded for birthday message (as self-reported at enrolment) 3. Tobacco and alcohol use recorded; participants who self-reported never using tobacco or alcohol were not sent messages about cutting down or quitting 4. Interactive-message to check timing and language of messages was acceptable (if not automated dialogue to change either language or timing) 5. Personalised timing of appointment reminders based on prospectively routinely collected computerised appointment data |
| **10.** | **Modifications during the trial** | Nil | | Nil |
| **11.** | **Planned intervention delivery** | Brief messages will be sent using an automated system independent of trial and clinical staff. Participants will be told that not everyone will be receiving the exact same messages. Participants will also be asked not to share their health messages with others. Intervention fidelity will be checked by confirming receipt at least of an initial “Welcome” message on their handset for all enrolled trial participants prior to randomisation. Message delivery reports will be monitored throughout the trial to check the intervention is being delivered as planned. Messages not delivered (network unavailable etc.) will be resent up to three times. Where message delivery fails for more than two weeks a failed message protocol will be initiated to track the participant and up-date their phone number if required. | | |
| **12.** | **Actual intervention delivery** | Will be reported once 12-month follow-up is complete | | |
